# Metastatic Inflammatory Myofibroblastic Tumor of the Spleen: A Case Report and Review of the Literature

**DOI:** 10.1155/2016/8593242

**Published:** 2016-11-28

**Authors:** Luca Koechlin, Andreas Zettl, Dieter Koeberle, Markus von Flüe, Martin Bolli

**Affiliations:** ^1^Department of Surgery, St. Claraspital, Basel, Switzerland; ^2^Department of Pathology, Viollier AG, Basel, Switzerland; ^3^Department of Oncology, St. Claraspital, Basel, Switzerland

## Abstract

*Introduction.* Inflammatory myofibroblastic tumors (IMT) of the spleen are rare neoplasms and only little is known about the origin and behavior of these tumors. Here we report the case of a 37-year-old woman with an atypical spindle cell neoplasm showing features strongly suggesting an IMT of the spleen with hepatic metastasis.* Methods.* A 37-year-old patient had been complaining about pain in the left upper abdomen for the last two months. A CT scan revealed a tumor mass in her spleen and liver. After complete staging, a splenectomy and atypical liver resection of segments VII and VIII were performed. Literature was screened for similar cases and existing further literature.* Results.* A R0 resection was achieved. Histological analysis showed a multinodular infiltration of the spleen by an atypical mesenchymal neoplasia. Immunohistochemically there was an expression of histiocytic markers (CD4, CD68) as well as smooth muscle cell markers (SMA, H-Caldesmon) in the tumor cells. A diagnosis of an atypical spindle cell neoplasm showing features most suggestive of an IMT was rendered.* Conclusion.* Synchronous hepatic metastasis of an IMT of the spleen is a rarity. Therefore no experience in the treatment of these tumors exists. Fibroblastic reticular cell tumor is a differential diagnosis, but differentiation of these two entities is difficult.

## 1. Introduction

Inflammatory myofibroblastic tumors (IMT) of the spleen are rare neoplasms. Because of their rarity only little is known about the origin and behavior of these tumors [1]. Here we report the case of a 37-year-old woman with a large multinodular mass in her spleen, in combination with liver metastasis with the histological pattern of an atypical spindle cell neoplasm showing features most suggestive of an IMT.

## 2. Case Presentation

A 37-year-old patient had complained about pain in the left upper abdomen for the last two months. The patient reported night sweats and an increase of the abdominal circumference but no fever or loss of weight. Initial blood analysis showed a microcytic hypochromic anemia with normal liver parameters. A CT scan in an external hospital revealed a tumor mass in the spleen and liver, primarily interpreted as a lymphoma. A transcutaneous needle biopsy was performed but was not conclusive.

**Figure 1:**
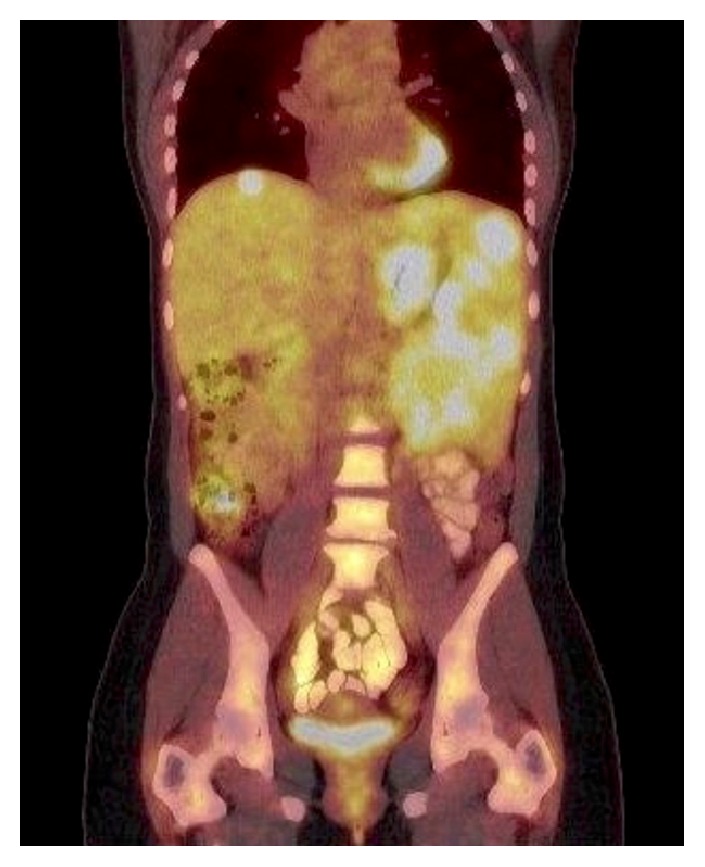
Preoperative PET-CT scan revealed multiple nodules in the spleen and a hepatic lesion with high metabolic activity.

PET-CT scan revealed multiple nodules in the spleen and a hepatic lesion with high metabolic activity. Moreover slightly PET-positive but not enlarged lymph nodes in the left axilla were detected (Figure 1).

The case was discussed at our interdisciplinary tumor board and the indication for surgery was given. A splenectomy and an atypical liver resection of segments VII and VIII were performed. There was no further examination of the axillary lymphadenopathy because it was clinically not manifest with nonpalpable lymph nodes and only weak FDG affinity without significant enlargement was shown. Intraoperatively the massively enlarged spleen showed a nodular surface and was attached to the tail of the pancreas. Two liver metastases were located intraoperatively by sonography. R0 resection was achieved and the postoperative course was uneventful. After two days in the intensive care unit the patient was discharged on the 10th postoperative day.

**Figure 2:**
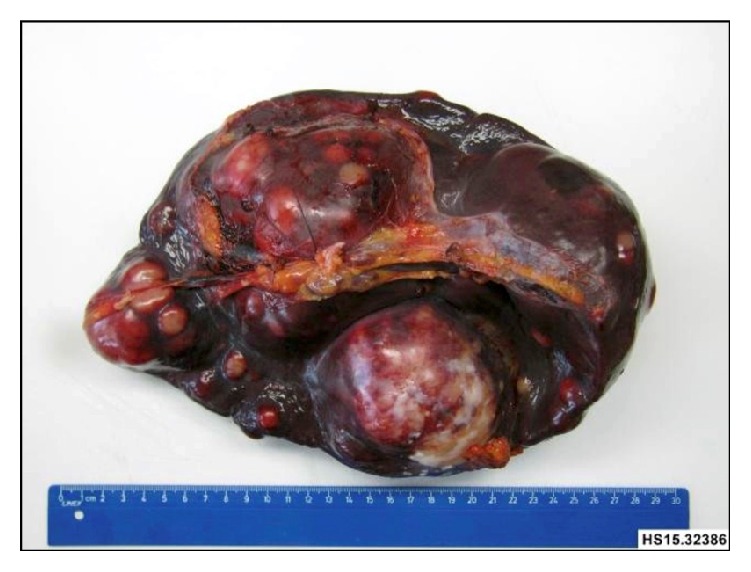
Macroscopically a multinodular infiltration of the spleen was seen.

**Figure 3:**
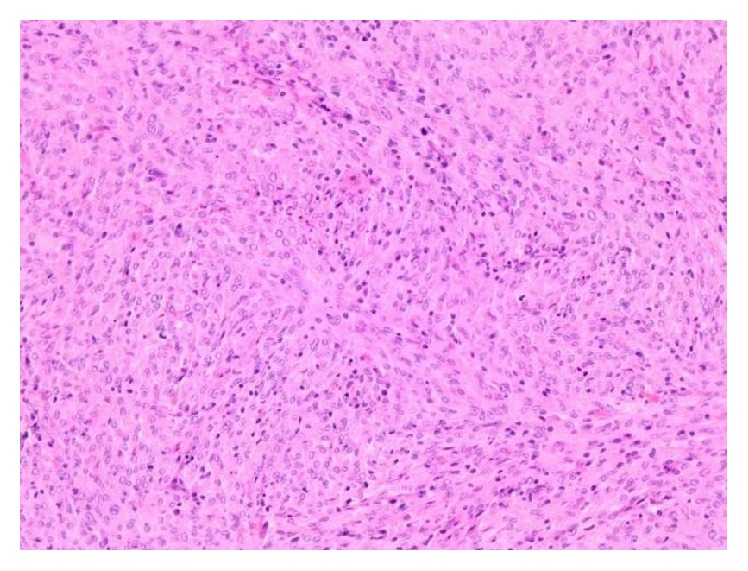
Histological analysis showed a multinodular infiltration throughout the spleen by atypical mesenchymal neoplasia.

Macroscopically the spleen was enlarged (measuring 22 × 13 × 10 cm and weighing 1825 g) and showed multiple well demarcated whitish nodules measuring up to 10,3 cm on the cut surface. The histological analysis showed a multinodular infiltration of the spleen by an atypical mesenchymal neoplasia. The tumor cells were spindled to polygonal with poorly demarcated eosinophilic cytoplasm. They grew in fascicular sheets and showed mitotic activity (up to × mitosis/10 high power fields) and focal punctuate necrosis. Intermingled between the tumor cells there was an inflammatory infiltrate of small lymphocytes (Figures 2 and 3).

Immunohistochemically the tumor cells showed an expression of the histiocytic markers (CD4 and CD68) as well as of smooth muscle cell markers SMA and H-Caldesmon. There was a weak partial positivity for CD34. Stains for dendritic cell markers CD21 and CD23, for pan-keratin AE1/3 and MNF116, and for S100, CD1a, CD117, ERG, DOG1, Smoothelin, Desmin, Melan A, HMB45, SOX10, HHV8, MDM2, D2-40, and STAT6 were negative. An Epstein-Barr virus association was not detected (negative EBER in situ hybridization). Furthermore, an expression of ALK1 or ROS1 was not detectable. The proliferation rate was 5–10% in Ki67 stain.

Considering all the results and after external consultation (kindly provided by C. Fletcher, Department of Pathology, Brigham and Women's Hospital, Boston, USA), a diagnosis of an atypical spindle cell neoplasm having features most suggestive of an inflammatory myofibroblastic tumor (IMT) was rendered.

Four weeks postoperatively, another PET-CT scan was performed. New metabolic lesions were detected in the mediastinum, the liver, and vertebral bones.

After two months of anti-inflammatory treatment with Ibuprofen 800 mg, another PET-CT scan showed a tumor progression with the appearance of a new hepatic metastasis in segment III and multiple bone as well as peritoneal metastases.

A targeted oncologic therapy was not available, due to a negative ALK and ROS1 expression. Therefore a palliative chemotherapy with weekly carboplatin and paclitaxel was started. According to a first restaging after 8 weeks of treatment, a partial remission was achieved. It was decided to continue chemotherapy for additional 2 to 4 months.

## 3. Discussion

In the final histological assessment the splenic tumor was labelled as an atypical spindle cell neoplasm having features most suggestive of an inflammatory myofibroblastic tumor of the spleen (IMT).

This is a cellular spindle cell neoplasm with an admixed chronic inflammatory infiltrate including both lymphocytes and plasma cells. The tumor cells have palely eosinophilic cytoplasm and mildly atypical plump or more tapering nuclei.

In “PubMed” database literature was screened for similar cases and existing literature.

Reviewing the currently available literature, only scarce information was found about IMT of the spleen. In most cases a benign course of the disease was observed. The WHO classification of soft tissue tumors considers this entity as intermediate malignant with a risk of metastatic spread below 5% [2]. In our case a synchronous hepatic and possible lymph node spread was already identified at the initial diagnosis.

To the best of our knowledge a synchronous hepatic metastatic spread of an IMT of the spleen is a rarity and is described in only one single report [3].

Furthermore, there seems to be a certain difficulty in distinguishing IMT of the spleen from fibroblastic reticulum cell (FBRC) tumor. This entity normally behaves more aggressively than IMT. However, there may be a possible overlap between both neoplasms. Martel et al. discussed a common origin of these two entities [4].

In our case greater cytologic atypia that one would normally expect in FBRC tumor was not detectable. Moreover, a FBRC tumor is almost invariably positive for keratin and/or desmin, whereas the tumor in our case was entirely negative for pan-keratin and/or desmin. Negativity for CD21 and CD35 also rules out the possibility of a follicular dendritic cell sarcoma.

The tumor in our case was also negative for ALK and ROS1. ALK positivity in inflammatory myofibroblastic tumors in patients above the age of 25 is relatively infrequent.

Because of its rarity no experience as for the treatment of these tumors exists. Beside surgical resection, given reasons for all other therapies are based on the published experience of single cases or small case series. Due to the diffuse progress in the first postoperative imaging, another surgical resection was no more an option. In literature there exist few reports of successful treatment of IMT with anti-inflammatory drugs [5, 6]. To the best of our knowledge none of these reports were about a splenic IMT. In our case, treatment with Ibuprofen 800 mg/day failed. However, we could induce a partial remission thereafter by using polychemotherapy.
